# Response Format, Not Semantic Activation, Influences the Failed Retrieval Effect

**DOI:** 10.3389/fpsyg.2019.00599

**Published:** 2019-04-04

**Authors:** Saeko Tanaka, Makoto Miyatani, Nobuyoshi Iwaki

**Affiliations:** ^1^ Department of Childhood Education, Tokushima Bunri University, Tokushima, Japan; ^2^ Department of Psychology, Hiroshima University, Hiroshima, Japan; ^3^ Faculty of Education, Iwate University, Iwate, Japan

**Keywords:** failed retrieval effect, semantic activation, retrieval-based learning, knowledge acquisition, learning promotion, response format, testing effect

## Abstract

In educational settings, tests are mainly used to measure the extent to which learners’ knowledge and skill have been acquired. However, the act of taking a test also promotes learning *itself*. In particular, making errors on tests (i.e., searching for erroneous information) promotes learning. This is called the “failed retrieval effect” (FRE) and has been the subject of considerable study. Previous research shows that enhanced learning does not occur if feedback correcting an error is delayed. This is attributed to the relative absence of activated information. In this study, we manipulated both the amount of information to be retrieved prior to learning and the delay time until feedback is given to investigate their effects on learning. As a result, even when multiple incorrect answers were given to increase the degree of semantic activation, learning was not promoted beyond that found with traditional procedures that rely on only one incorrect response. The timing of feedback (immediate, short-delay, long-delay) also did not impact FRE. However, the manipulation of response format for erroneous information resulted in degraded performance when responses were typed and feedback was delayed. Based on this result, we suggested that the failed retrieval effect was not affected by semantic activation at the time of retrieval but was affected by response format. Moreover, the processing necessary for typing may affect FRE under the delayed feedback condition.

## Introduction

In educational settings, tests are implemented to evaluate the extent to which learners’ knowledge and skills have been acquired; in the context of memory research, recall and recognition tests are used to evaluate performance. However, taking tests itself also promotes learning. When initial testing is conducted on the material to be memorized, performance on subsequent tests improves, beyond what is learned through re-reading this material. This phenomenon, variously called the “testing effect” or “retrieval-based learning,” has generated an extensive literature (e.g., Dempster, 1996; Roediger and Karpicke, 2006a,b; Putnam and Roediger, 2013; Smith et al., 2013; Carpenter and Yeung, 2017; Sundqvist et al., 2017). Notably, enhancing memory *via* testing, contrary to both learners’ ordinary sense and behavioral learning theory, is not limited to when students answer correctly; it also occurs when they answer incorrectly. This is known as the “failed (unsuccessful) retrieval effect (FRE)” or the “pretesting effect.”

Kornell et al. (2009) conducted an experiment on FRE using paired association tasks involving weakly associated words. For example, the cue word, “tide,” is weakly related to the target word, “beach” in that the cue evokes the target in less than 5% of people sampled. In Kornell et al. (2009), participants learned word pairs under either a retrieval condition (test) or a control condition (read-only), after which a cued-recall final test was given. In the retrieval condition, an initial test was given in which a target word was answered based on a cue word, and then the word pair to be memorized was presented (correct feedback). In the control condition, the word pair was presented without an initial test. Because participants did not know which word was paired with the cue word before the initial test, they were unable to answer with the correct target word (i.e., failed retrieval) for almost 95% of words. However, in the final test, cued-recall performance for word pairs was significantly higher in the retrieval condition as compared to the control condition.

FRE occurs for a variety of stimuli and participants, including word pairs (e.g., Kornell et al., 2009; Grimaldi and Karpicke, 2012; Hays et al., 2013; Yan et al., 2014); short phrases or sentences (Richland et al., 2009; Kornell, 2014); word pairs of unfamiliar English words and their definitions or foreign vocabulary and their translations (Potts and Shanks, 2014); and trivia questions with multiple choice answers in children (Marsh et al., 2012). However, learning promotion does not occur equally for all stimuli; the type of stimulus and the timing of the correct answer feedback interact. In other words, although no effect occurs when word pairs are used as a stimulus without immediate feedback (e.g., Experiment 3 in Kornell et al., 2009; Grimaldi and Karpicke, 2012; Experiment 1 in Kornell, 2014) and when sentences such as trivia questions are used, learning is promoted even when the correct answer feedback is delayed (by about 1 day) (e.g., Experiment 3 in Richland et al., 2009; Kornell, 2014).

Why do such interactions occur? The answer is the FRE mechanism. Grimaldi and Karpicke (2012) conducted three experiments using word pairs as stimuli. In Experiment 1, they compared learning outcomes of a weakly related word pair (as in Kornell et al., 2009) and a completely unrelated word pair (e.g., “pillow—leaf”). In Experiment 2, in the initial test, they compared the conditions under which participants were free to answer the target word and conditions under which the experimenter presented a similar word called a “lure.” In Experiment 3, researchers compared the condition in which feedback was given immediately after the initial test and the condition in which the correct answer feedback was presented after performing the initial test with all word pairs (i.e., delayed feedback). In Experiment 1, FRE occurred only when there was an association between cue and target. In Experiment 2, performance was poorer in when the experimenter presented the word than in the control condition. In Experiment 3, learning promotion did not occur in either the delayed feedback or control conditions, which was a different result compared to the immediate feedback condition. Based on these results, Grimaldi and Karpicke (2012) explained the FRE mechanism as follows. First, participants freely retrieve target words from the cues in the initial test, thereby activating a semantic trace network (*search set*). At the time of the initial test, “candidates” (Grimaldi and Karpicke’s term for words that are likely to be associated with the cue) are given as a response, but the activation spreads to other candidates, including the correct target word. The activation eventually promotes learning of the presented correct answer feedback. Additionally, results of Experiment 3 showed that this activation lasted only for a short time, as in priming.

Conversely, Kornell (2014, Experiment 3a) found the wrong answer learning promotion effect, even when corrective feedback was delayed. In Kornell’s experiment, participants were asked to respond to trivia questions such as “Who was *Time Magazine*’s ‘Man of the Year’ in 1938?” and were given the correct feedback 24 h later. Participants were unable to correctly answer 86% of the questions, and these questions were analyzed. When a final test was conducted 24 h after the provision of feedback (i.e., 48 h after answering the questions), a learning promotion effect occurred that was attributed to the initial test, despite the delayed feedback. Based on these results, Kornell stated that there is no difference between FRE using word pairs in the immediate feedback condition and FRE using sentences in the delayed feedback condition, in which enhanced learning occurs because of retrieval. However, in the case of delayed feedback, it was likely that long-term memory had been activated concerning questions and incorrect answers. From the research of Grimaldi and Karpicke (2012), it appears that word-level information activated by FRE is not maintained with delayed feedback; however, for sentences, the learning benefits of FRE are obtained with delayed feedback (Kornell, 2014). The source of this discrepancy between studies is attributed to the amount of information activated. More information is activated when sentences are presented as a stimulus than when word pairs are given.

Thus, if we activate more information, will enhanced learning occur even with delayed feedback procedures using word pairs? Several studies have investigated the impact of the amount of activated information on the testing effect and FRE at the time of retrieval. For example, in Experiment 3a–5 by Lehman and Karpicke (2016), in the initial test conducted after participants had learned word pairs (i.e., in a testing effect procedure), they were asked to generate several words semantically associated with the cue words that differed from the target word they were supposed to remember (e.g., “mediator” words). As a result, on a final test in which participants were required to recall target words from cue words, their performance declined as the number of mediators increased. Regarding FRE, in Experiment 2 of Grimaldi and Karpicke (2012), as mentioned above, if the lure word presented by the experimenter was learned in addition to the to-be-remembered word pair, performance declined more than in the control condition. Moreover, in a recent study, Vaughn et al. (2017) used trivia questions and set the response interval between the question presentation and the correct feedback presentation to either 0 s (i.e., questions and answers were presented together, without allowing for retrieval) or between 5 and 30 s. As a result, although the correct answer rates on the initial test increased with longer retrieval times (Vaughn and colleagues believe that the participants continued to think of correct answers, and thus, more semantic revitalization occurred), “whether or not retrieval occurred” had an effect on the performance in the final test, while length of time did not.

From these studies, it appears that differences in the amount of activated information may not have a significant impact on learning promotion. However, Lehman and Karpicke (2016) asked participants to generate and learn words that were different from previously remembered target words. Furthermore, in Grimaldi and Karpicke (2012), lure words were presented by experimenters and not by the participants themselves. Therefore, it may be possible that this produced interference. Moreover, in Vaughn et al. (2017), sentences were used as stimuli; in light of Kornell’s (2014) results, it is also conceivable that sufficiently rich information to promote retrieval was activated, even if only for a short period. According to Grimaldi and Karpicke (2012), the search set theory explaining FRE is based on search-related theories, such as the SAM model. In the SAM model (Raaijmakers and Shiffrin, 1981; Gillund and Shiffrin, 1984), difficulty in recalling the learned item is dependent on the amount of related information. It is believed that as the amount of information related to the item to be learned increases, the recall possibility of that item in the final test increases. Furthermore, although word pair and sentence stimuli were compared by Kornell (2014), it is possible that the underlying characteristics of these stimuli may also differ greatly (i.e., the ease or difficulty of memorization, etc. may affect results).

In this study, we use word pairs to consider the relationship between delayed feedback and the richness of the semantic network activated at the time of initial testing. We investigate the impact of the amount of information retrieved in the initial test on FRE, particularly whether learning is not enhanced under delayed feedback as shown in previous studies or if it is affected by the amount of erroneous information retrieved in the initial testing. With reference to the SAM model and Vaughn et al. (2017), the semantic network activated was set using the number of words retrieved in the initial test.

In this study, we compared the impact of different retrieval conditions, achieved by adjusting feedback delay and the degree of semantic richness in the initial test, to promote learning. The retrieval conditions were determined based on the number of words answered in the initial test (one or two words) and the length of time to feedback (immediate feedback or delayed feedback). The four experimental conditions were (1) single word immediate feedback, (2) multiple word immediate feedback, (3) single word delayed feedback, and (4) multiple word delayed feedback. Participants were asked to learn word pairs under five conditions consisting of these four and a control condition just to read and remember the word pairs. Furthermore, unlike in previous studies (e.g., Grimaldi and Karpicke, 2012), participants read the responses aloud during the initial test rather than typing responses. This was done to reduce individual differences affecting learning outcomes, as there is large variation in typing accuracy and speed of Japanese college students.

Previous studies found no differences between oral responses and typed responses on testing effects (Putnam and Roediger, 2013). However, there has been no research on the effect of response format on FRE itself. Regarding the relationship between the response format and memory, for example in the production effect (MacLeod et al., 2010), whispering, typing, or handwriting items promote learning, but learning is promoted most by reading aloud (Forrin et al., 2012). While reading aloud, auditory processing occurs in addition to motor processing. Additionally, compared to whispering, reading aloud results in a stronger auditory signal, which requires more active encoding. Furthermore, Pinet and Nozari (2018) demonstrated that, unlike verbal responses, typed responses require post-lexical processing. Considering this information, if this study fails to produce the same results as the previous ones, it may possibly be due to the influence of response format (verbal vs. typed response) on FRE.

This study considers the following five hypotheses:

1. Final test performance is better under the condition in which subjects retrieve a word from a cue they think *might* be the target word (hereinafter “retrieved word”), before immediately being presented with the correct target word.
2. Learning will be promoted if feedback on the retrieved answer is given immediately, but not if it is delayed. This hypothesis is based on the findings of Grimaldi and Karpicke (2012) as well as Kornell (2014), particularly under the condition in which participants were asked to answer with only a single retrieved word (i.e., in which the activation of semantic networks is not rich).
3. If learning is promoted when considerable information is recalled from a cue even when feedback is delayed (Kornell, 2014), then learning promotion will occur when participants answer using multiple retrieved words even when feedback with the correct target words is delayed.
4. Performance on a final test with immediate feedback, in which participants are asked to give multiple retrieved words, will be better than performance in a typical procedure (i.e., in which participants are asked to answer using a single retrieved word with immediate feedback).
5. Performance on a final test will be affected by response format. Auditory processing occurs spontaneously and post-lexical processing is not required, so performance may be more improved when spoken than typed. Moreover, verbal response causes auditory processing but does not require post-lexical processing, which may benefit the oral response format.

## Experiment 1

All experiments in this study were approved by the Ethical Review Committee of Tokushima Bunri University.

### Materials and Methods

#### Participants

A mix of 27 university and junior college students (10 men and 17 women with an average age of 19.9 years, *SD* = 1.2) participated in the experiment and were paid an honorarium of 1,000 yen. Participants were treated in accordance with the APA Ethical Guidelines. Participants’ native language was Japanese, and each participant learned word pairs under all four experimental and one control conditions described below.

#### Materials

Stimuli were a hundred Japanese word pairs, consisting of a cue word and a target word (e.g., cheese-pizza, motorcycle-tire, the underlined word is the target). (All stimuli are provided in the Supplementary Material and were selected based on Mizuno, 2011). Word pairs were selected so that their association strength (i.e., the rate at which the target word would be guessed as the first response to the cue word) was between 0.041 and 0.054—that is, fairly weak (Kornell et al., 2009). Cue words were three morae in Japanese notation; target words were between two and four morae, and word notations included Chinese-derived characters (*kanji*) as well as both Japanese syllabaries (*hiragana* and *katakana*).

#### Conditions

**Figure 1:**
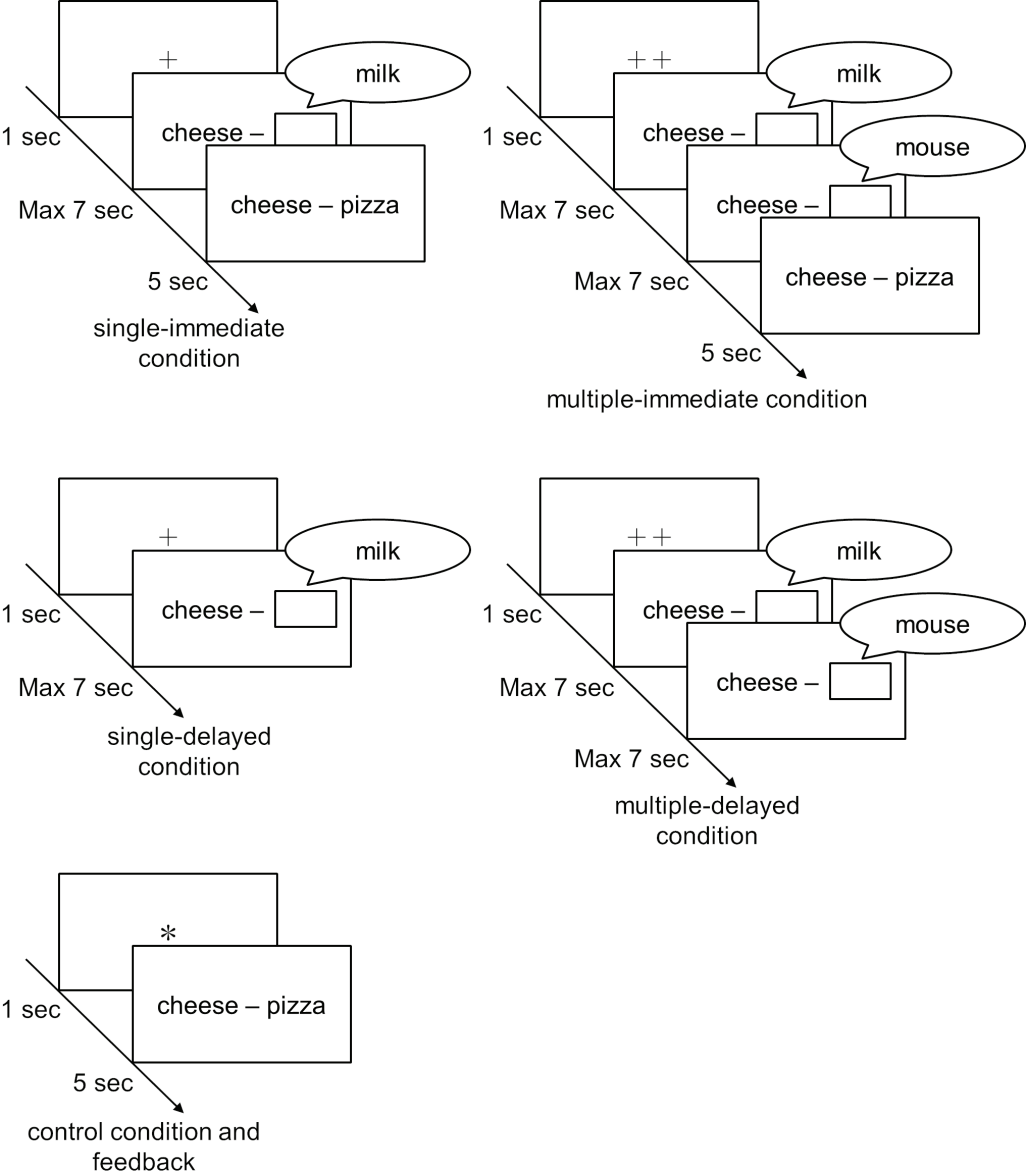
Procedures for each condition at the word learning stage.

There were four experimental and one control conditions. Participants were exposed to one-answer and two-answer conditions (respectively, the single condition and multiple condition) in which they answered one or two words that *might* be target words (“retrieved words”). Additionally, conditions were established in which the correct answer (the target word to be recalled) was provided as feedback immediately after the initial test and in which feedback was delayed (respectively, the immediate condition and the delayed condition). The five overall conditions, in combination, were as follows: the single-immediate condition, multiple-immediate condition, single-delayed condition, and multiple-delayed condition, as well as an additional control condition that did not involve answering a retrieved word (Figure 1).

In the single-immediate condition, a plus symbol (+) was shown for 1 s on a computer screen as a fixation point. Participants were taught that the number of plus symbols indicated the number of words to be answered (retrieved words). Then, a cue word and a frame were shown on the screen for 7 s, during which participants were asked, based on the cue word, to say as quickly as possible a single word that might be the target word. Once a participant answered, the experimenter hit the ENTER key to proceed to the next screen, on which the correct target word was displayed for 5 s. If a participant was unable to answer within the 7-s time window, the display automatically moved to the next screen (which displayed the correct target word in the single-immediate condition). Participants were instructed to memorize the cue and target word pairs.

In the multiple-immediate condition, two plus symbols were shown for 1 s, after which, just as in the single-immediate condition, a cue word and a frame were shown on the screen for 7 s. Then, participants were asked to say a retrieved word as quickly as possible. When the participant answered, the experimenter hit the ENTER key to proceed to the next screen; when 7 s elapsed without the participant answering, the display automatically moved to the next screen. In this multiple-immediate condition, the procedure was repeated twice to make participants say two words (i.e., participants were requested to say another retrieved word after again being shown a cue word and frame for 7 s, proceeding to the next screen once 7 s had elapsed or they said a word). After answering, as in the single-immediate condition, the correct target word for the cue word was shown for 5 s, and participants were instructed to memorize the cue and target word pairs.

In the single-delayed condition, the initial test was the same as in the single-immediate condition. As a point of contrast, after a participant had answered a word or 7 s had elapsed, the screen shifted to the next trial immediately, without providing the correct target word from the previous cue as feedback. Feedback was presented in the same way as under the control condition (described below) once a certain number of trials were completed.

In the multiple-delayed condition, the initial test was the same as in the multiple-immediate condition, and feedback was presented in the same way as under the control condition once a certain number of trials were completed.

For the control condition, an asterisk (*) was displayed for 1 s as a fixation point, after which both the cue word and target word were displayed for 5 s, without implementing the initial test. Participants were asked to memorize the word pairs. These conditions were each allocated 20 word pairs, and the allocation of word pairs was counterbalanced.

#### Procedure

**Figure 2:**
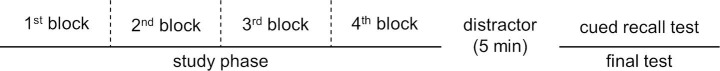
Overall flow of Experiment 1.

**Figure 3:**
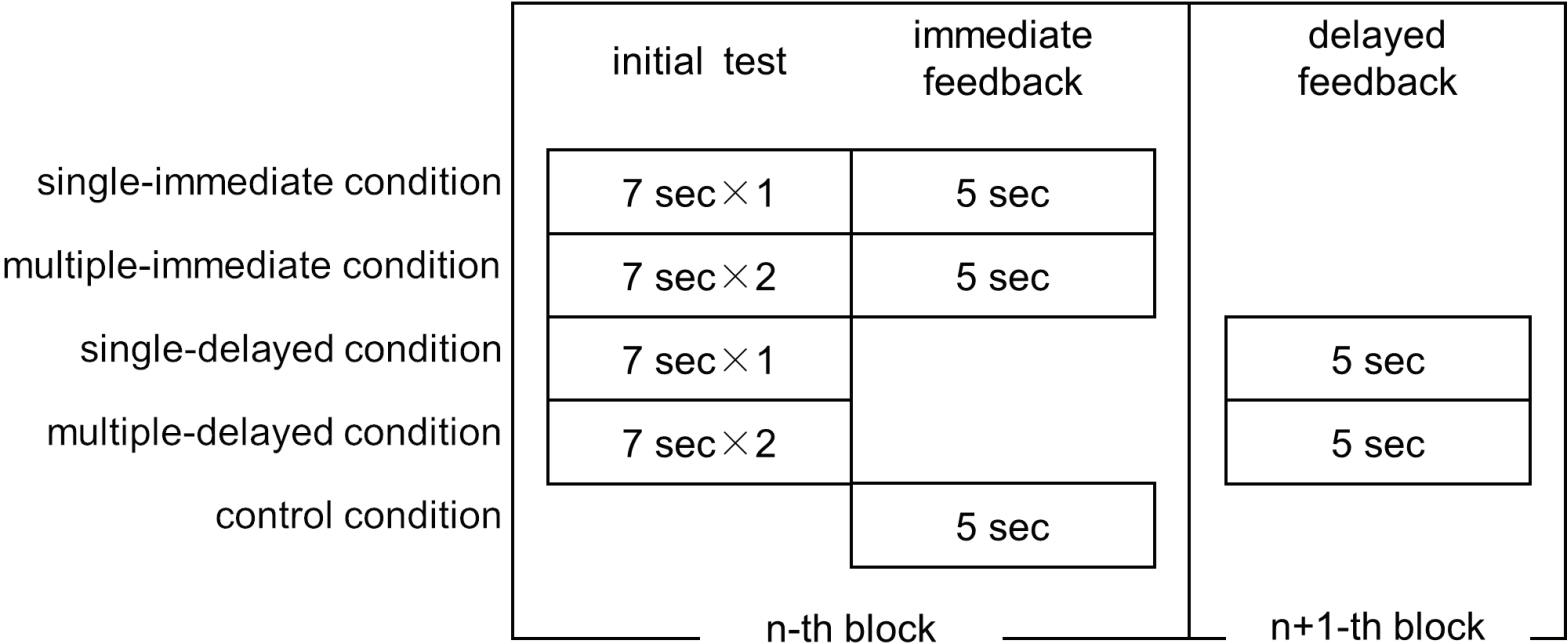
Initial test and feedback implementation order for each block in each learning condition.

This experiment consisted of three phases, the study phase (initial tests and feedback of word pairs), a distractor task, and a final test (cf. Kornell et al., 2009). However, to delay feedback, the study phase was divided into four blocks (e.g., Hays et al., 2013) (Figures 2 and 3). In other words, in the single- and multiple-delayed conditions, the correct target word feedback for word pairs presented in one block was given in the subsequent block. No break was provided between blocks, and participants were not told that the study phase was divided into blocks. In the delayed conditions, the time from cue word presentation to target word presentation ranged between 0.4 and 11.6 min (*M* = 5.6 min, *SD* = 2.4 min).

In each block, five pairs of words were assigned to each condition, with a total of 25 word pairs presented in random order. In addition, in the second and subsequent blocks, 35 pairs [25 trials and 10 feedback trials under the two delayed conditions presented in the previous block (five trials respectively)] were presented in random order. Ten word pairs assigned to the delayed conditions in the fourth block were presented using the same procedure as in the immediate conditions; these were excluded from the analysis. In addition, a total of 15 word pairs (10 trials assigned to the immediate conditions and 5 trials assigned to the control condition) learned in the first block were also excluded from the analysis in order to equate the number of trials in the delayed conditions and the lag up to the final test.

After obtaining participants’ written consent, instructions were given about the learning strategies for each condition in the study phase. Afterwards, a practice session was conducted using 10 word pairs not used in the experimental session to check whether participants understood the instructions. Thereafter, participants were asked to learn word pairs using the five conditions described earlier: the single-immediate condition, multiple-immediate conditions, single-delayed condition, multiple-delayed condition, and control condition. The order in which word pairs were presented in the blocks was randomized for each participant.

In the distractor phase, participants spent 5 min on a mental arithmetic task (performing arithmetic calculations involving two- and three-digits). Mathematical formulae and frames were presented on a computer screen, whereupon participants were instructed to enter answers into the frame using a numeric keypad.

The final test was a cued-recall task. After a plus symbol was displayed as a fixation point on the screen for 1 s, a cue word and frame were displayed for 7 s, during which time participants were instructed to recall and say the correct target word (not retrieved word) paired with the cue word. The correct answer was not displayed even when participants could not recall the target word in time or answered incorrectly. The cued-recall task in the final test followed the same procedure for all learning condition, and the order in which cue words were presented was randomized for each participant.

### Results and Discussion

The trials were analyzed after excluding a total of 25 word pairs assigned to the single-immediate, multiple-immediate, and control conditions in the first block and the single- and multiple-delayed conditions in the fourth block. In addition, any word pairs answered correctly in the initial test (4%) were excluded. Cohen’s *d* and partial *η* ^2^ were used as the effect size of the *t*-test and analysis of variance, respectively.

When comparing final test performance in the control condition to that in the four retrieval conditions (pooled performance of the four retrieval conditions), the correct answer rate was higher for word pairs assigned to the retrieval condition. Consistent with previous research, we found that prior retrieval of erroneous information promoted learning correct information (retrieval condition *M* = 0.75, *SD* = 0.14, control condition *M* = 0.62, *SD* = 0.21, *t*(26) = 4.11, *p* < 0.01, *d* = 0.73).

**Figure 4:**
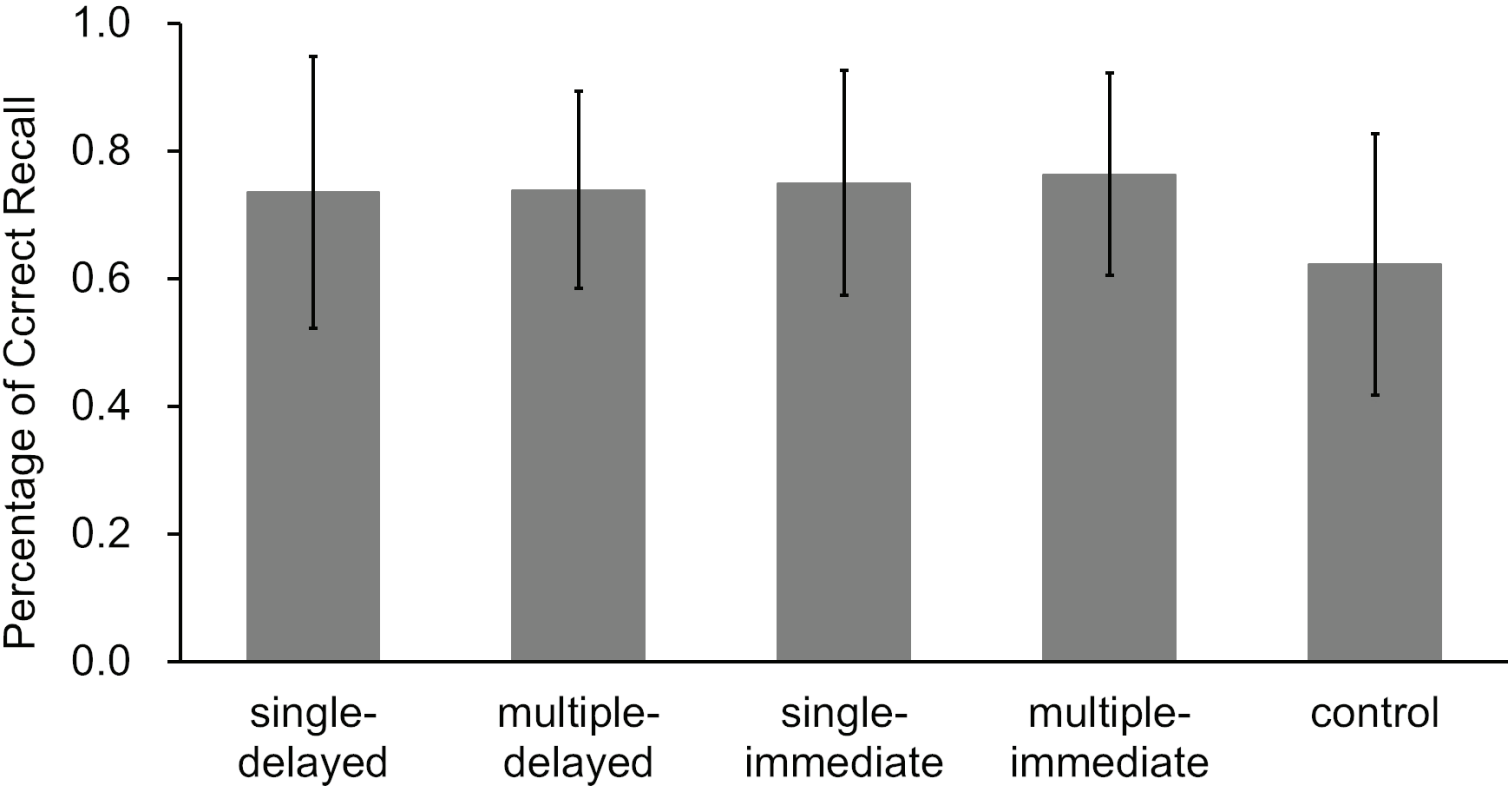
Correct answer rates on the final tests for each learning condition in Experiment 1 (error bars represent *SD*).

A two-factor analysis of variance using two (presence or absence of delay) by two (number of retrieved words answered) for the four retrieval conditions showed no main effects of delay (*F*(1, 26) = 0.39, *p* = 0.54 $\eta_{p}^{2}$ = 0.02) or number of answers (*F*(1, 26) = 0.09, *p* = 0.77 $\eta_{p}^{2}$ < 0.01). The interaction of delay and number of retrieved words answered was not significant [*F*(1, 26) = 0.04, *p* = 0.83 $\eta_{p}^{2}$ < 0.01] (Figure 4). Detailed results of each condition are described in Supplementary Material.

Therefore, the learning was promoted by conducting retrieval in the initial test, consistent with previous studies. Although the presentation time of the target word was the same in the retrieval and control conditions, the presentation time of the cue word was different. There is a possibility that this may have influenced the FRE. However, in previous studies (Kornell et al., 2009; Grimaldi and Karpicke, 2012), the presentation time of the cue word in the initial test was as 7–8 s, and the presentation time of the feedback (i.e., a pair of cue and correct target) was 5 s, as in this experiment. Similar results were obtained in these experiments. Moreover, in the study by Kornell et al. (2009), the effect of FRE did not change even if the stimulus presentation times between conditions were equal, that is, even when the presentation time of the control condition was increased. Thus, our results may have been influenced by the specifics of processing in the retrieval condition and not by the difference in the presentation time of cue words.

In contrast to previous studies, we found improved learning even when correct answer feedback was delayed. Grimaldi and Karpicke (2012) suggested that learning promotion by failed retrieval does not occur under conditions where feedback is delayed, so semantic networks that activate for only a short time like priming exist as a failed retrieval mechanism. However, the results of this experiment did not support this conclusion. Notably, encouraging the activation of the semantic networks by increasing the number of words to be answered had no impact on the FRE. Thus, the difference between sentences and word pair stimuli cannot be fully explained by “the richness of semantic networks” (Kornell, 2014).

However, in this experiment, the time interval to feedback varied greatly, from just under a minute to just under 12 min. Although the procedure in this experiment followed that of previous studies in which the effect of failed retrieval disappeared due to delayed feedback, it is possible that the variation in time until feedback may have influenced learning, and perhaps the delay time was not long enough. Accordingly, in Experiment 2, we minimized the variation of delay time to examine the impact of the relative length of the interval until feedback in promoting learning.

## Experiment 2

In Experiment 2, we manipulated the interval of time until feedback to be either 4–5 min (short-delay condition) or 10–11 min (long-delay condition). This allowed us to investigate the effect and length of feedback delay on learning.

### Materials and Methods

#### Participants

A total of 25 native Japanese speakers comprising a mix of university and junior college students (5 men and 20 women with an average age of 19.3 years, *SD* = 2.0) participated in the experiment and were paid an honorarium of 1,000 yen.

#### Materials

One hundred sets of Japanese word pairs consisting of a cue word and a target word were used, as in Experiment 1.

#### Conditions

We set four learning conditions. Under the three retrieval conditions, participants were presented with correct answer feedback set at 0 min (i.e., immediately), randomly varying from 4 to 5 min (short-delay) or randomly varying from 10 to 11 min (long-delay) after retrieving erroneous information. There was also a control condition in which retrieval did not take place.

The immediate condition was exactly the same as the single-immediate condition in Experiment 1. The short-delay and long-delay conditions were almost the same as the single-delayed condition in Experiment 1, with the sole difference being the feedback delay time. After the initial test of each stimulus, feedback was presented after the set time had elapsed. Thirty-one word pairs were allocated to the control and immediate conditions, 20 word pairs to the short-delay condition, and 18 word pairs to the long-delay condition. Allocations to each stimulus condition were counterbalanced. Final tests of all words learned in the study phase were performed, but analysis was restricted to 18 word pairs randomly selected from the word pairs assigned to each condition. The word pairs that were not analyzed were filler tasks to ensure appropriate timing in delay conditions.

#### Procedure

As in Experiment 1, there were three phases: the study phase (an initial test and feedback), a distractor, and a final test. The study phase was divided into six blocks.

**Figure 5:**
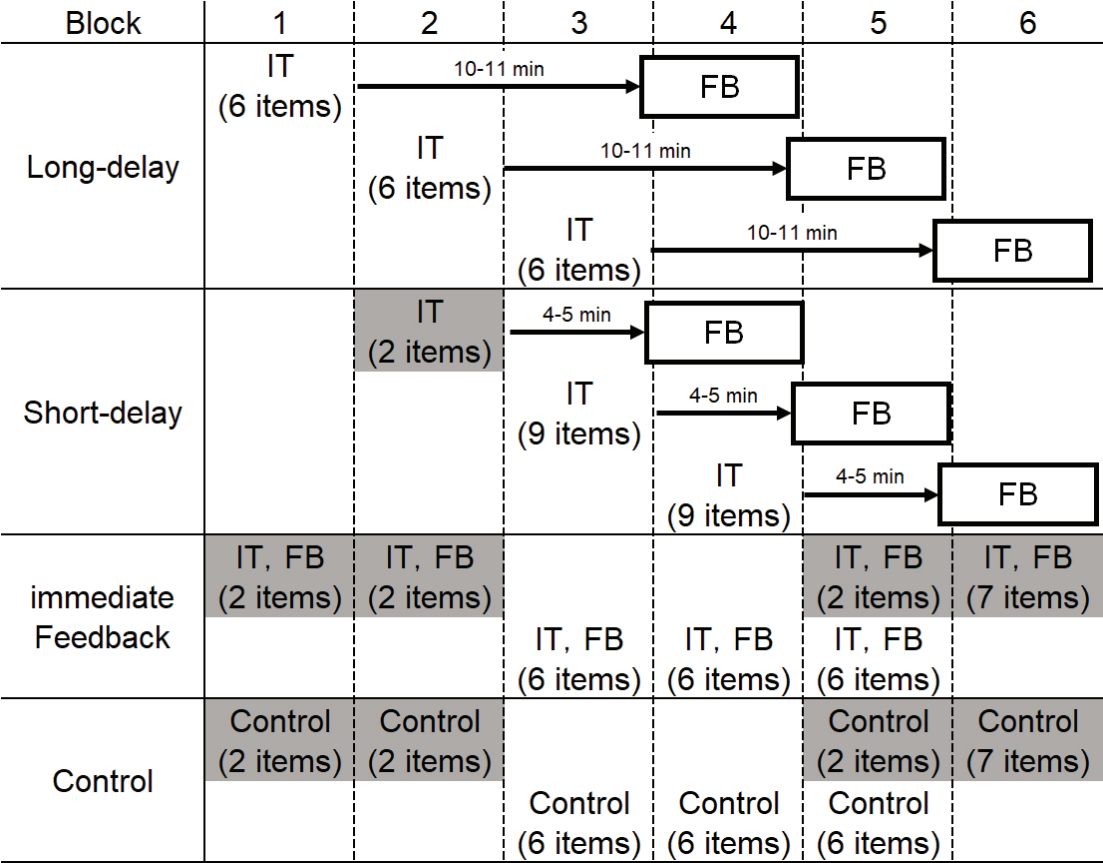
Stimulus presentation flow for each condition in Experiment 2. Stimuli for each condition were allocated to Blocks 1–6. In the long-delay and short-delay conditions, feedback was given after intervals of 10–11 and 4–5 min, respectively. We set filler trials (grayed-out cells) for the following reasons: (1) in order to ensure the delay time before feedback presentation was for long- and short-delay conditions (10–11 or 4–5 min) and (2) participants were not made aware that the program was divided into blocks. IT indicates “initial test” and FB indicates “feedback.”

Stimulus presentation flow for each condition in Experiment 2 is shown in Figure 5. The initial tests of the long-delay condition were implemented in Blocks 1–3. The initial tests of the short-delay condition were implemented in Blocks 2–4. In long- and short-delay conditions, feedback was presented after 10–11 min or 4–5 min following the initial test, respectively. In addition, the initial tests and feedback for immediate conditions and control conditions were implemented in Blocks 1–6. As mentioned above, we analyzed 18 items in each condition and the other 28 items were used in filler trials.

After obtaining written consent, participants practiced the study phase, learned word pairs, and then completed the distractor task and final test, in that order.

### Results and Discussion

As in Experiment 1, all trials were analyzed, with the exception of the 28 word pairs in the filler trials, and the word pairs answered correctly in the initial test (2%).

The correct answer rate was higher for word pairs assigned to the retrieval condition as compared to this in the control condition; furthermore, the retrieval of erroneous information promoted the learning of correct information (retrieval condition *M* = 0.70, *SD* = 0.16, control condition *M* = 0.56, *SD* = 0.16, *t*(24) = 7.38, *p* < 0.01, *d* = 1.48).

**Figure 6:**
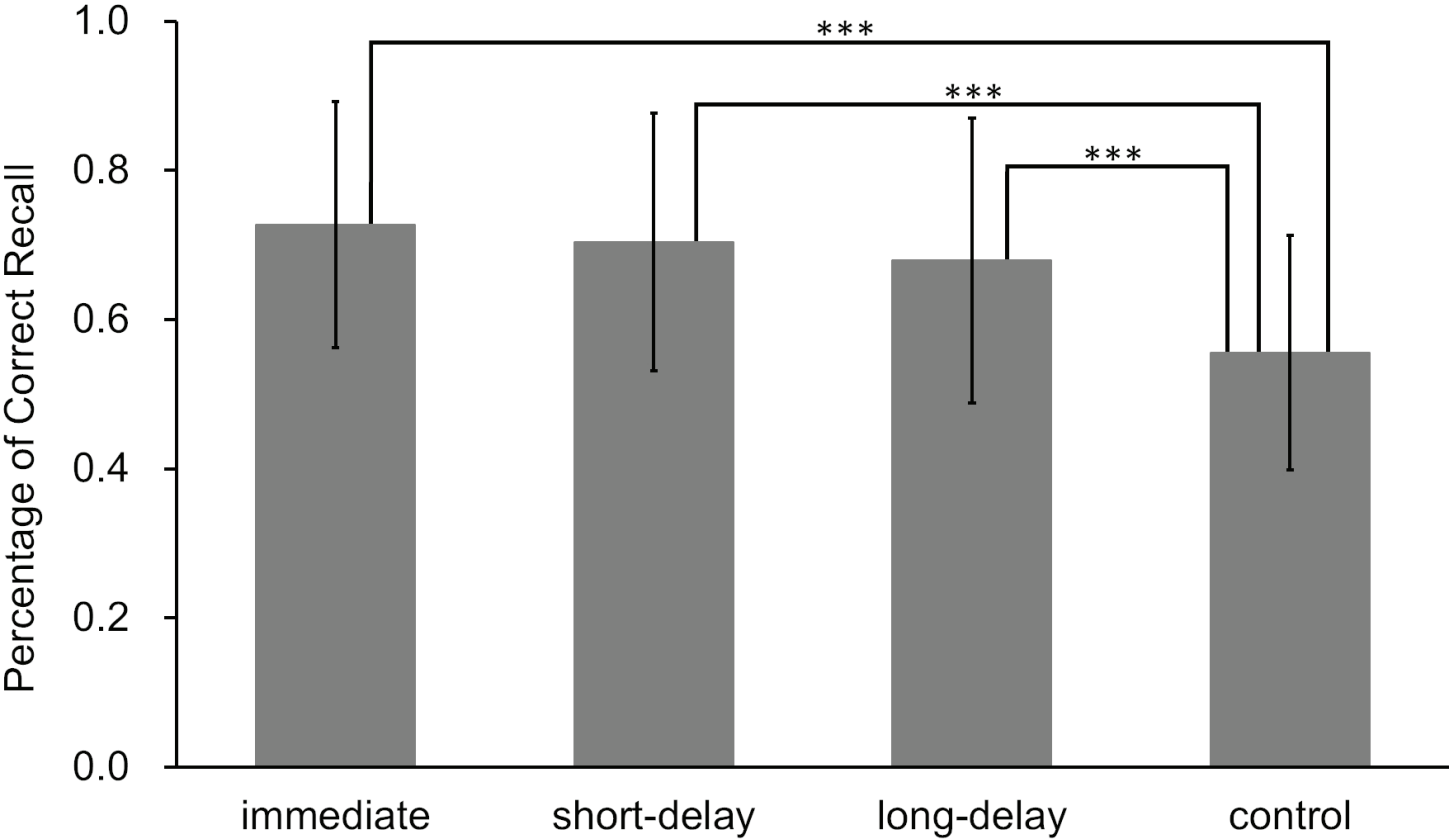
Final test performance for each learning condition in Experiment 1 (error bars represent *SD*); a one-factor analysis of variance revealed significant differences between each of the conditions connected by a straight line (***p < 0.01).

A one-factor analysis of variance of final test performance for the four word-pair learning conditions yielded a significant main effect (*F*(3, 72) = 17.03, *p* < 0.01, $\eta_{p}^{2}$ = 0.42; Figure 6). After multiple pairwise comparisons, we found significant differences between the control condition and each of the three retrieval conditions (*ps* < 0.01). Although there were no significant differences between retrieval conditions, the correct answer rate was highest for the immediate condition (*M* = 0.73, *SD* = 0.16), followed by the short-delay condition (*M* = 0.70, *SD* = 0.17) and the long-delay condition (*M* = 0.68, *SD* = 0.19). In addition, the effect size of multiple comparisons was *d* = 0.14 for the difference between immediate and short-delay conditions, *d* = 0.27 for the difference between immediate condition and long-delay conditions, and *d* = 0.13 for the difference between short-delay and long-delay conditions.

Thus, learning was again promoted even when correct answer feedback was delayed, unlike in previous studies (e.g., Grimaldi and Karpicke, 2012; Kornell, 2014). Moreover, this remained unchanged even when using a longer delay time for feedback. Although not statistically significant, there was a trend for performance to decrease over time. That is, if the delay time was extended, it is possible that final test performance would decline further.

Moreover, in Experiments 1 and 2, participants orally answered the retrieved word. The response format does not affect the result in the testing effect procedure (Putnam and Roediger, 2013). However, auditory processing or post-lexical processing may affect FRE.

Given these considerations, in Experiments 3a and 3b, we further extended the delay time and investigated the effect of retrieved word response formats on FRE.

## Experiment 3

In Experiment 3, we investigated whether Experiments 1 and 2 results could be reproduced when extending the delay time, as well as whether FRE differed depending on the retrieved word response format – i.e., by having participants answer verbally (Experiment 3a) or by typing (Experiment 3b).

### Experiment 3a

#### Materials and Methods

##### Participants

A total of 25 native Japanese speakers comprising a mix of university and junior college students (3 men and 22 women with an average age of 19.6 years, *SD* = 1.4) participated in the experiment and were paid an honorarium of 1,000 yen. Each participant learned word pairs under five conditions.

##### Materials

One hundred sets of Japanese word pairs consisting of a cue word and a target word were used, as in Experiment 1.

##### Condition

As in Experiment 1, we set five conditions consisting of a single-immediate condition, multiple-immediate condition, single-delayed condition, multiple-delayed condition, and control condition. Word pairs were presented in each condition using the same method as in Experiment 1.

##### Procedure

In Experiment 3, the study phase was divided into two blocks in order to provide a delay time. In the first block, we implemented the initial test for the delayed conditions. In the second block, we implemented the feedback for the delayed conditions, the initial test and feedback for the immediate conditions, and the presentation of the control condition. The second block began 15 min after the start of the first block. After the completion of the first block, participants were given Tetris to play until the start of the second block (play time varied for each participant depending on how long it took them to complete the first block). The actual delay time ranged between 15.1 and 25.2 min (*M =* 20.4 min, *SD* = 0.5).^1^ The distractor and the final test were implemented using the same method as in the previous experiments.

#### Results and Discussion

Because no filler trials were provided for this experiment, only trials where the retrieved word and target word were identical (5%) were excluded from analysis.

When comparing final test performance for the control and retrieval conditions, the correct answer rate was higher for word pairs for which retrieval took place; the retrieval of erroneous information promoted the learning of correct information (retrieval condition *M* = 0.68, *SD* = 0.16, control condition *M* = 0.52, *SD* = 0.20, *t*(24) = 5.96, *p* < 0.01, *d* = 0.88).

**Figure 7:**
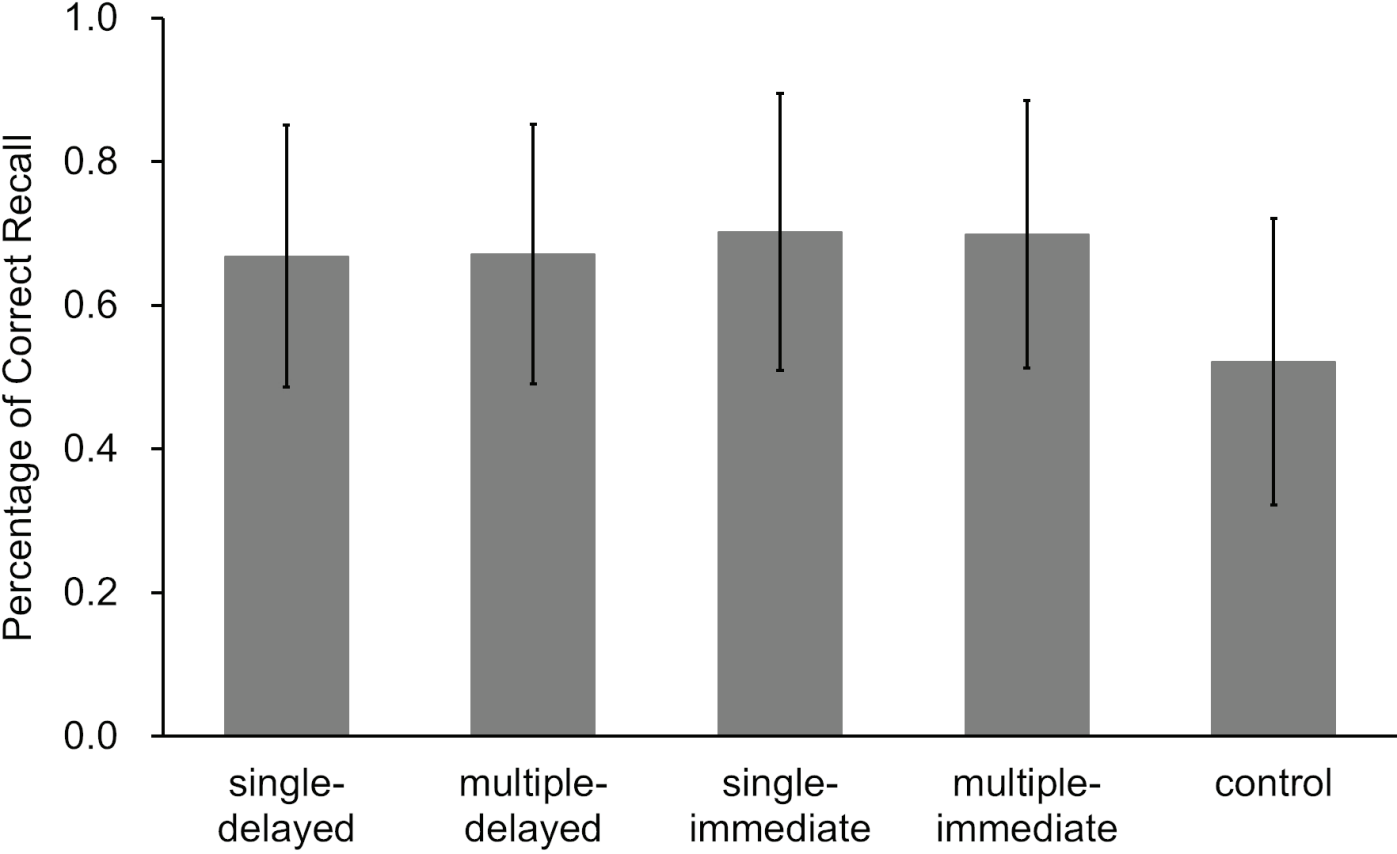
Correct answer rates on the final tests for each learning condition in Experiment 3a (error bars represent *SD*).

Results of a two-factor analysis of variance using two (presence or absence of delay) by two (number of words answered) for the retrieval conditions revealed no significant main or interacting effects. The effect of delay was *F*(1, 24) = 1.34, *p* = 0.26, $\eta_{p}^{2}$ = 0.05; of words answered was *F*(1, 24) < 0.01, *p* = 0.98, $\eta_{p}^{2}$ < 0.01; and of the interaction was *F*(1, 24) = 0.03, *p* = 0.87, $\eta_{p}^{2}$ < 0.01 (Figure 7). Results clearly showed that learning was promoted when participants spoke retrieved words aloud in the same way as before, even when correct answer feedback was delayed by an average of 20 min.

### Experiment 3b

#### Materials and Methods

##### Participants

A total of 25 native Japanese speakers comprising a mix of university and junior college students (2 men and 23 women with an average age of 20.0 years, *SD* = 1.2) participated in the experiment and were paid an honorarium of 1,000 yen. Each participant learned word pairs under five conditions.

##### Materials and Conditions

Materials and conditions used in this experiment were the same as in Experiment 3a.

##### Procedure

The retrieved word response format in the initial test was changed to typing rather than speaking; otherwise, the procedure was identical to Experiment 3a. Participants used a keyboard that did not allow them to use character conversion keys (when inputting Japanese text on a keyboard, participants type using the Roman alphabet and convert to *kanji* characters as necessary. For this experiment, conversion to *kanji* was not done because of time limitations). Rather, participants were asked to type retrieved words in the on-screen text box using alphabetical input (after input, the alphabet was automatically converted to *kana* characters, which were displayed on the monitor). Participants were asked to type retrieved words as quickly as possible during the answer interval (7 s) and instructed to proceed to the next trial by hitting the ENTER key after they had finished typing. The actual delay time for the delayed conditions ranged between 15.1 and 25.3 min (*M* = 20.0 min, *SD* = 0.5).

#### Results and Discussion

As in Experiment 3a, only trials where the retrieved and target words were identical (4%) were excluded from analysis.

When comparing final test performance for the control condition and retrieval conditions (pooled performance of the four conditions), the correct answer rate was higher for word pairs assigned to retrieval conditions. In this experiment, as in others in this study, retrieval of erroneous words promoted the learning of target words (retrieval condition *M* = 0.66, *SD* = 0.15, control condition *M* = 0.57, *SD* = 0.18, *t*(24) = 3.87, *p* < 0.01, *d* = 0.54).

In addition, as a result of two-factor analysis of variance using two (presence or absence of delay) by two (number of words answered) for the retrieval conditions, the main effect of delay (*F*(1, 24) = 6.19, *p* = 0.02, $\eta_{p}^{2}$ = 0.21) and the interaction of delay and the number of answers (*F*(1, 24) = 9.24, *p* < 0.01, $\eta_{p}^{2}$ = 0.28) were significant, while the main effect of number of answers (*F*(1, 24) = 0.11, *p* = 0.74, $\eta_{p}^{2}$ = 0.01) was not significant.

**Figure 8:**
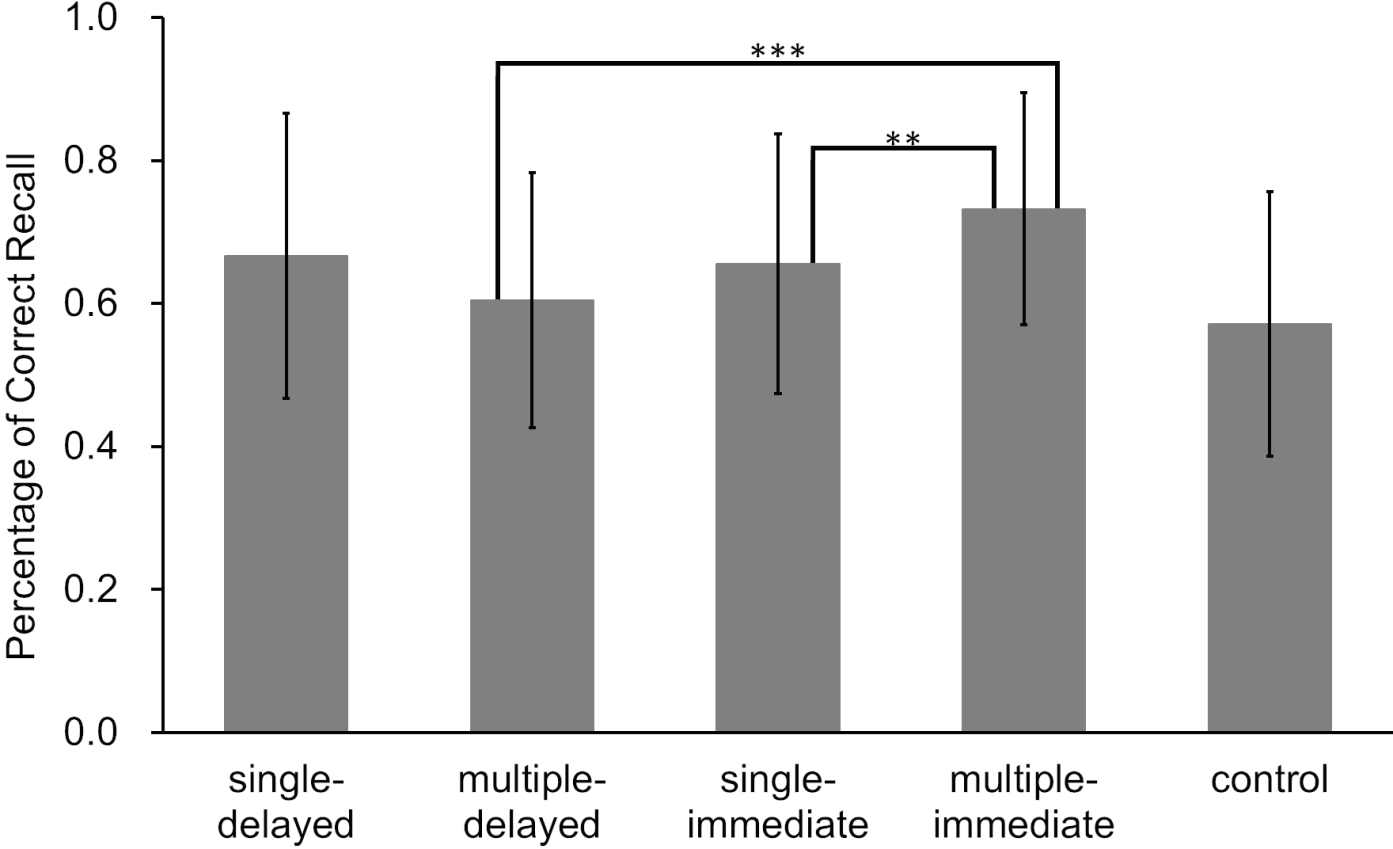
Correct answer rates on the final tests for each learning condition in Experiment 3b (error bars represent *SD*). The results of a two-factor analysis of variance on the four retrieval conditions revealed significant differences between each condition, connected by a straight line (***p < 0.01, **p < 0.05).

As this interaction was significant, we conducted a simple main effect test. This showed that the effect of delay under the multiple answer conditions (*p* < 0.01, $\eta_{p}^{2}$ = 0.43) and the effect of number of retrieved words answered under the immediate conditions (*p* = 0.02, $\eta_{p}^{2}$ = 0.20) were significant. The effect of the number of retrieved words answered under the delayed feedback conditions was not significant (*p* = 0.07, $\eta_{p}^{2}$ = 0.13) (Figure 8).

In the typed response conditions, the significant main effect of delay indicated learning was facilitated more with immediate feedback condition as compared to delayed feedback, as in previous studies. Also, when given immediate feedback, there was the possibility that the correct answer rate would improve when multiple answers were required. However, when multiple words were recalled, performance in the delayed feedback condition was lower than in the immediate feedback condition. In the following section, we address differences in response format of retrieved words (verbal or typing) on FRE.

## General Discussion

One of the goals of this study was to determine the impact on FRE of the amount of information to be retrieved. We investigated whether we could compensate for the nullifying effect of delayed feedback on learning shown in previous studies by increasing the degree of semantic network activation. In Experiments 1 through 3a, in which participants said retrieved words aloud, final test performance did not improve in the immediate feedback conditions even when semantic networks were activated more intensively. That is, response differences between the single-immediate and multiple-immediate conditions were minimal. Nor did the amount of information to be retrieved have any effect in the delayed feedback conditions. Unlike in previous studies, learning was promoted even when feedback was delayed.

When we calculate feedback delay time from previous studies (e.g., Grimaldi and Karpicke, 2012, Experiment 3), the delay seems to be about 12 min at most.^2^ Additionally, Kornell (2014, Experiment 1) used word pairs as the stimulus, and the time lag in the delayed feedback condition was about 4 min, on average. In the current study, the delay was longer, so it cannot be that the influence of the delay that should have originally occurred disappeared.

However, when the retrieved word was typed (vs. spoken), we replicated previous study results; that is, the delay condition reduced the recall performance compared to the immediate condition. From this, we surmised that the response format of word retrieval influenced FRE, especially at delayed feedback. A study on the production effect (Forrin et al., 2012) demonstrated that reading items aloud improves learning more than writing or whispering. The explanation for this is that auditory processing promotes information encoding. So, it may be important to encode erroneous information at the time of the initial test to promote FRE learning. In our Experiments 1–3a, it is possible that information encoding was promoted when participants said their answers aloud. On the other hand, typing responses might not be sufficient for encoding erroneous information, as shown in Experiment 3b.

Another consideration is the role of attention. Unlike verbal reactions, typing requires processing after determining the response words (Pinet and Nozari, 2018). Although not limited to typing, Rapp and Fischer-Baum (2014) explained cognitive processing needed to spell. First, based on input information, we access semantic representations in long-term memory. Next, the orthographic representation derived from long-term memory is processed with the orthographic working memory system (Graphemic Buffer). When spelling, we continue to activate orthographic information, so that letters can be written in the correct order (Rapp and Fischer-Baum, 2014). In our study, the orthographic working memory system was required only in the typed response condition. Therefore, there may have been additional attentional demands on working memory in the typed response format as compared to the spoken word format.

Although retrieval is processed preferentially when attention is divided and is unaffected even when performing a dual task in parallel, the encoding and elaboration processes are inhibited under dual tasks (Craik et al., 1996; Mulligan, 2008). In the testing effect, the retrieval process not affecting attentional distribution is considered important, as it does not decrease performance even if a dual task is performed at the time of initial testing (Buchin and Mulligan, 2017). However, the impact of dual task performance on FRE procedures has not been considered. From the viewpoint of attention, there is a possibility that the encoding process is necessary for FRE.

Based on this information, our study results can be explained as follows. When there was immediate post-retrieval feedback, learning was facilitated regardless of whether or not erroneous information was encoded. Conversely, feedback was delayed, and it was necessary to keep erroneous information activated until correct answer feedback was presented. Therefore, in the easy-to-encode oral response condition (Experiment 1–3a), there was no difference in performance between the immediate and delayed feedback conditions. However, we found an interaction in the more difficult-to-encode typed response format in Experiment 3b. When multiple typed answers were required and feedback was delayed, retrieved words were not sufficiently encoded; retrieved words, the target word, and other semantic networks activated during the initial test might have been mixed-up, so the correct word could not be supplied. On the other hand, when multiple typed answers were required and feedback was immediate, the more likely those retrieved words (erroneous information) were encoded, and thus, the learning outcome was improved.

As with FRE, the hypercorrection effect concerns “learning through error.” Erroneous answers for which learners have high confidence are more likely to be corrected with feedback compared to erroneous answers for which they have low confidence. In the final test, if participants could remember the initial error in a stimulus, the correct recall rate of that stimulus was higher (Butler et al., 2011). This also suggests that the encoding of erroneously retrieved words is important in the context of FRE.

Grimaldi and Karpicke (2012) and Kornell (2014) consider the activation and richness of semantic networks (in other words, processing until responding with erroneous information) important in the context of FRE. However, present study results show that the truly important factor is that erroneous information is encoded and then held until correct answers are presented (Butler et al., 2011; see also Iwaki et al., 2017). This suggests that, for example, when using learners’ errors to consolidate learning, the most important consideration is how to process incorrect information rather than an emphasis on “generating errors.”

In this study, we manipulated the response format between participants. The relationship between feedback delay and the number of words to be answered was used as a within-participant variable. If the response format was also a within-participant variable, the number of stimuli per condition would be too small, allowing only 10 trials. However, it is possible that the between-subjects design influenced our findings. Future should also remove the “number of words to answer” factor, which had little effect on final testing, and instead make response format a within-participants factor. In addition, it will be necessary to recall the words retrieved during the initial test at the time of feedback or final test in order to directly investigate whether recalling erroneous information directly promotes learning. Furthermore, in order to examine the possibility that attention was affected when participants typed responses, it may be necessary to retest using a general dual task, following Buchin and Mulligan (2017).

This study indicates that when learners say incorrect responses aloud (meaning that encoding is sufficient), learning is facilitated by correct feedback given immediately or following short or long delays. On the other hand, when responding with an error while typing (meaning that information was not encoded sufficiently), it is better to provide correct feedback as soon as the learner has generated an error. Moreover, if it is clear that it is impossible to provide correct feedback immediately, it is better for the learner to participate in a process that makes it easier to encode the error (e.g., to respond aloud or to prevent divided attention). In addition, the size of the semantic network activated has little effect on learning promotion.

As both FRE and the testing effect are learning methods that make use of “tests,” which are usually used in educational settings, their introduction into educational settings will not incur a cost (Roediger and Pyc, 2012). Moreover, both FRE (Tanaka and Miyatani, 2015), and the testing effect (Brewer and Unsworth, 2012) promotes learning regardless of working memory capacity, which is closely related to individual learning proficiency. Along with correct answers on tests, if methods and mechanisms for effectively utilizing test errors to promote learning become clearer, educators can strategically use error on tests to instruct students. Moreover, learners will be able to take advantage of their own errors. As a result, it will eventually lead to the development of enhanced self-education ability.

## Ethics Statement

This study was carried out in accordance with the recommendations of the Ethical Review Committee of Tokushima Bunri University with written informed consent from all subjects. All subjects gave written informed consent in accordance with the Declaration of Helsinki. The protocol was approved by the the Ethical Review Committee of Tokushima Bunri University.

## Author Contributions

ST contributed to the data collection and analysis and drafted the manuscript. MM and NI contributed to the revision of the experiment procedure and the manuscript.

### Conflict of Interest Statement

The authors declare that the research was conducted in the absence of any commercial or financial relationships that could be construed as a potential conflict of interest.

## Supplementary Material

The Supplementary Material for this article can be found online at: https://www.frontiersin.org/articles/10.3389/fpsyg.2019.00599/full#supplementary-material

## Footnotes

^1^In Experiment 3, it was necessary to present the feedback of the word pair learned in block 1 (delayed feedback condition) evenly and randomly in all other conditions during block 2. Therefore, the delay time was set to about 10 minutes.

^2^In this experiment, initial tests of all 60 words are performed in random order, and feedback is presented at random in subsequent blocks. Therefore, if feedback for a cue word that appears at the end of the initial test block comes out at the beginning of a feedback block, the lag is thought to be shorter.
