# Supplementary material for: Response Format, Not Semantic Activation, Influences the Failed Retrieval Effect
Source: Front Psychol. 2019 Apr 4;10:599. doi: 10.3389/fpsyg.2019.00599 (PMC6459060; doi:10.3389/fpsyg.2019.00599)
Supplement: Supplementary file 1 [file Data_Sheet_1.pdf]

Supplementary Material

Response Format, Not Semantic Activation, Influences the Failed Retrieval Effect

Saeko Tanaka*, Makoto Miyatani, Nobuyoshi Iwaki

*** Correspondence:** Saeko Tanaka: saekot2356@gmail.com

Supplementary Table 1. List of stimuli used in this study. During the experiments, we presented Japanese words at the top of each cell, with their English translation shown in parentheses below them.

cue word	target word	cue word	target word	cue word	target word
赤字 (deficit)	借金 (debt)	世論 (public opinion)	世間 (society)	カエル (frog)	雨 (rain)
移民 (immigration)	難民 (refugees)	論理 (logic)	理屈 (reason)	カルタ (karuta)	遊び (play)
汚染 (pollution)	川 (river)	いかだ (raft)	遭難 (distress)	ゲスト (guests)	招待 (invitation)
親子 (parent and child)	関係 (relationship)	いのち (life)	人間 (human)	コイン (coin)	スロット (slot)
会議 (conference)	討論 (debate)	うどん (udon)	きつね (fox)	コラム (column)	本 (book)
価格 (price)	商品 (product)	えくぼ (dimple)	頬 (cheek)	サウナ (sauna)	熱 (heat)
科学 (science)	進歩 (progress)	おかず (side dish)	弁当 (box lunch)	シルク (silk)	高級 (high class)

火災<br>(fire)	炎<br>(flame)	おとこ<br>(man)	筋肉<br>(muscle)	スパイ<br>(spy)	敵<br>(enemy)
家庭<br>(home)	円満<br>(amicable)	おなか<br>(stomach)	背中<br>(back)	スリル<br>(thrill)	映画<br>(movies)
看護<br>(nurse)	医療<br>(medical)	おむつ<br>(diapers)	子供<br>(child)	チーズ<br>(cheese)	ピザ<br>(pizza)
管理<br>(management)	かぎ<br>(key)	かかと<br>(heel)	くつ<br>(shoes)	テスト<br>(test)	期末<br>(term-end)
機械<br>(machine)	精密<br>(precision)	かたち<br>(form)	積み木<br>(building block)	テニス<br>(tennis)	運動<br>(motion)
苦労<br>(hardship)	労働<br>(labor)	かばん<br>(bag)	通学<br>(go to school)	テラス<br>(terrace)	家<br>(house)
検査<br>(inspection)	健康<br>(health)	さかな<br>(fish)	うろこ<br>(scales)	ドレス<br>(dress)	女性<br>(woman)
権利<br>(right)	国民<br>(people)	せりふ<br>(dialogue)	演劇<br>(theater)	ネオン<br>(neon)	夜景<br>(night view)
古典<br>(classic)	昔<br>(old days)	たばこ<br>(tobacco)	害<br>(harm)	バイク<br>(bike)	タイヤ<br>(tire)
資格<br>(qualification)	英検<br>(eiken)	だるま<br>(dharma)	選挙<br>(election)	バイト<br>(part-time job)	時給<br>(hourly wage)
刺激<br>(stimulation)	感覚<br>(sense)	だんご<br>(dango)	くし<br>(comb)	パイプ<br>(pipe)	水道<br>(water supply)
資源<br>(resource)	有限<br>(limited)	たんす<br>(chests of drawers)	ひきだし<br>(drawer)	バケツ<br>(bucket)	リレー<br>(relay)
思考<br>(thought)	停止<br>(stop)	つくえ<br>(desk)	教室<br>(classroom)	パズル<br>(puzzle)	難解<br>(esoteric)

自信 (confidence)	喪失 (loss)	とうふ (tofu)	豆 (beans)	ビール (beer)	大人 (adult)
自然 (nature)	森林 (forest)	となり (next)	友達 (friend)	ビデオ (video)	映像 (image)
時代 (age)	戦国 (Sengoku)	にきび (acne)	青春 (youth)	ポンプ (pump)	空気 (air)
深夜 (late night)	徘徊 (loitering)	のれん (goodwill)	布 (cloth)	マイク (microphone)	音声 (voice)
心理 (mind)	学問 (learning)	はかま (hakama)	着物 (kimono)	ミイラ (mummy)	死体 (cadaver)
世界 (world)	国 (country)	はさみ (scissors)	刃物 (knife)	ミシン (sewing machine)	ぬいもの (sewing)
危険 (danger)	安全 (safety)	はんこ (penchant)	名前 (name)	モグラ (mole)	地中 (underground)
知識 (knowledge)	頭脳 (brain)	ふすま (bran)	たたみ (tatami)	モデル (model)	ファッション (fashion)
手紙 (letter)	メール (email)	ふとん (futon)	羽毛 (down)	モラル (moral)	マナー (manner)
電話 (phone)	会話 (conversation)	まつり (festival)	花火 (fireworks)	ラジオ (radio)	エフエム (FM)
道具 (tool)	工具 (industrial tool)	まぶた (eyelid)	瞳 (pupil)	ランチ (lunch)	ご飯 (rice)
道路 (road)	交通 (traffic)	りんご (apple)	青森 (Aomori)	ワイン (wine)	フランス (France)
野菜 (vegetables)	果物 (fruit)	わいろ (bribe)	悪 (evil)		

Supplementary Table 1. Final test results of each learning condition in Experiment 1.

	Mean	*SD*
control	0.62	0.21
single-delayed	0.74	0.21
multiple- delayed	0.74	0.15
single-immediate	0.75	0.18
multiple-immediate	0.76	0.16
result of pooling retrieval conditions	0.75	0.14

Supplementary Table 2. Final test results of each learning condition in Experiment 2.

	Mean	*SD*
control	0.56	0.16
immediate	0.73	0.16
short-delay	0.70	0.17
long-delay	0.68	0.19
result of pooling retrieval conditions	0.70	0.16

Supplementary Table 3. Final test results of each learning condition in Experiment 3a.

	Mean	*SD*
control	0.52	0.20
single-delayed	0.67	0.18
multiple- delayed	0.67	0.18
single-immediate	0.70	0.19
multiple-immediate	0.70	0.19
result of pooling retrieval conditions	0.69	0.16

Supplementary Table 4. Final test results of each learning condition in Experiment 3b.

	Mean	*SD*
control	0.57	0.18
single-delayed	0.67	0.20
multiple- delayed	0.61	0.18
single-immediate	0.66	0.18
multiple-immediate	0.73	0.16
result of pooling retrieval conditions	0.66	0.15
